# Modeling of intracranial tumor treating fields for the treatment of complex high-grade gliomas

**DOI:** 10.1038/s41598-023-28769-9

**Published:** 2023-01-30

**Authors:** David J. Segar, Joshua D. Bernstock, Omar Arnaout, Wenya Linda Bi, Gregory K. Friedman, Robert Langer, Giovanni Traverso, Sumientra M. Rampersad

**Affiliations:** 1 Department of Neurosurgery, Brigham and Women’s Hospital, Harvard Medical School, Boston, MA USA; 2 David H. Koch Institute for Integrative Cancer Research, Massachusetts Institute of Technology, Cambridge, MA USA; 3 Department of Pediatrics, University of Alabama at Birmingham, Birmingham, USA; 4 Department of Mechanical Engineering, Massachusetts Institute of Technology, Cambridge, MA USA; 5 Division of Gastroenterology, Brigham and Women’s Hospital, Harvard Medical School, Boston, MA USA; 6 Department of Physics, University of Massachusetts, Boston, MA USA; 7 Department of Electrical and Computer Engineering, Northeastern University, Boston, MA USA

**Keywords:** CNS cancer, Computational biophysics, Cancer in the nervous system

## Abstract

Increasing the intensity of tumor treating fields (TTF) within a tumor bed improves clinical efficacy, but reaching sufficiently high field intensities to achieve growth arrest remains challenging due in part to the insulating nature of the cranium. Using MRI-derived finite element models (FEMs) and simulations, we optimized an exhaustive set of intracranial electrode locations to obtain maximum TTF intensities in three clinically challenging high-grade glioma (HGG) cases (i.e., thalamic, left temporal, brainstem). Electric field strengths were converted into therapeutic enhancement ratios (TER) to evaluate the predicted impact of stimulation on tumor growth. Concurrently, conventional transcranial configurations were simulated/optimized for comparison. Optimized intracranial TTF were able to achieve field strengths that have previously been shown capable of inducing complete growth arrest, in 98–100% of the tumor volumes using only 0.54–0.64 A current. The reconceptualization of TTF as a targeted, intracranial therapy has the potential to provide a meaningful survival benefit to patients with HGG and other brain tumors, including those in surgically challenging, deep, or anatomically eloquent locations which may preclude surgical resection. Accordingly, such an approach may ultimately represent a paradigm shift in the use of TTFs for the treatment of brain cancer.

## Introduction

Non-invasive electric brain stimulation known as tumor treating fields (TTF) offers a novel approach to combating tumor growth in a myriad of cancers and as such has been described as a fourth treatment modality alongside surgery, chemotherapy and/or radiation^1^.

The first FDA-approved indication for TTF was obtained in a sub-type of high-grade glioma (HGG) (i.e., glioblastoma [GBM]) with this tumor type representing the most common primary brain cancer in adults^2^. Of note, Novocure’s Optune^®^ TTF device was initially approved for the treatment of recurrent supratentorial GBM^3,4^ and later for use as maintenance therapy in combination with temozolomide^5^.

In TTF use scalp electrodes to emit a continuous alternating electric field that creates an intracellular dipole moment1 during the formation of the mitotic spindle in dividing cells, resulting in a dose-dependent inhibition of tumor growth^6–9^. As TTF only inhibits tumor growth during stimulation, efficacy is significantly improved with strict patient compliance (i.e., with the prescribed protocol suggesting that the device be worn for at least 18 h per day)^10^. Of note, both preclinical and clinical evidence suggest that increased field strength within the tumor and increased stimulation time improve TTF efficacy^6–8,10^. Accordingly, developing solutions to achieve these clinically relevant parameters is of critical importance.

One barrier to increasing field strengths via a transcutaneous/calvarial approach is the attenuation of field strength by the skull itself; modeling of transcranial current stimulation and measurements in human cadavers have shown that up to 75% of injected currents do not reach the brain^11,12^. Therefore, bypassing the skull is an appealing solution if one is seeking to achieve clinically relevant increases in field strengths within brain tumors. Consistent with this line of thinking, a study utilizing targeted craniectomies in conjunction with TTF to bypass the skull was conceptualized and has shown promising results both in silico and in a phase I clinical trial^13^. It is prudent to note that while this method may provide an avenue for substantially higher field strengths in hemispheric tumors, modeling suggests that achieving therapeutic field strengths in deep brain targets remains unlikely^14^.

Accordingly, we hypothesized that neurosurgically implanted intracranial stimulation system(s) may provide substantial improvements in the treatment of HGGs/GBM by both increasing the strength of the electric fields delivered and the duration of the therapy. Here we provide an initial proof of concept of the benefits and challenges of optimizing intracranial TTF for three clinically challenging cases using realistic finite element head models. We used two measures to analyze the results of the simulations: electric field strength and therapeutic enhancement ratio (TER) in the region of interest (ROI) encompassing the volume of the tumor.

## Results

**Figure 1:**
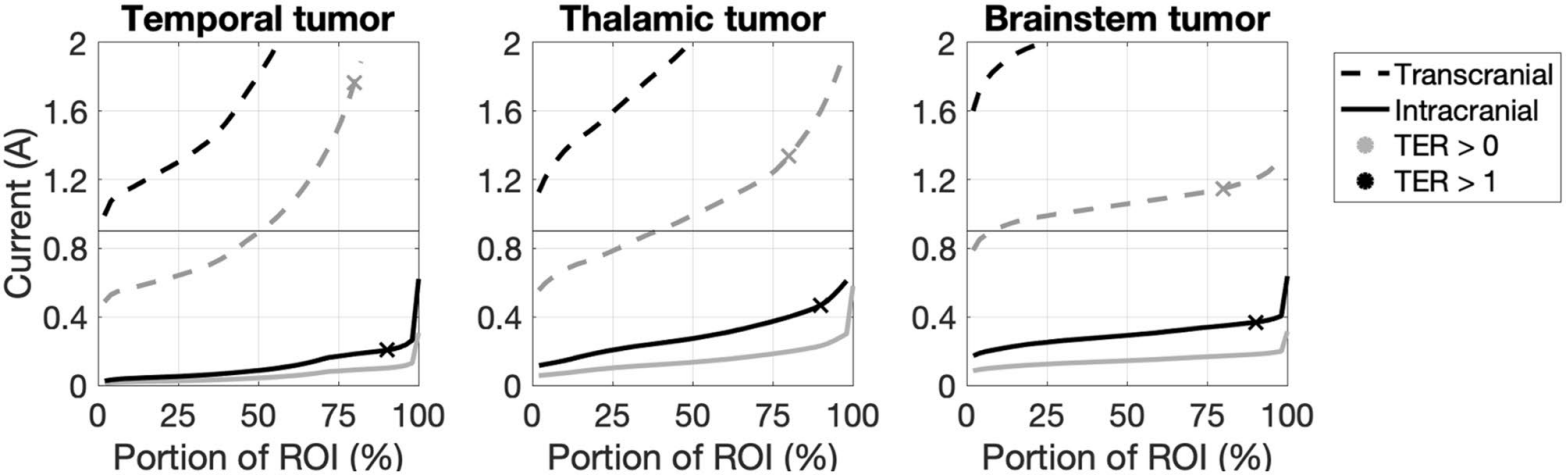
TER optimization curves. Each curve indicates the amount of current needed to achieve a TER greater than 0 (gray) or 1 (black) in a percentage of the ROI for transcranial (dashed) and intracranial (solid) TTF for three tumor cases. Horizontal lines indicate the current level conventionally used for transcranial TTF (0.9 A). Crosses indicate configurations that were selected as the optimal results, which are visualized in Fig. 2 and Supplementary Fig. S3.

We performed an optimization over 17 transcranial and 5050 intracranial configurations to identify those that required minimum current to achieve $\mathrm{TER}>0$ or $\mathrm{TER}>1$ within the ROIs for the three tumor cases presented. The TER quantifies the effects of an electric field on a tumor: TER values above 0 indicate a reduction in tumor growth, TER of 1 corresponds with complete growth arrest, and TER values above 1 indicate tumor shrinkage. Complete proliferation arrest (TER of 1) occurs in glioma cell cultures when fields reach 2.25 V/cm in the treated tumor^6^ and this value has been used as a benchmark in other studies^14^. We therefore calculated the percentage of the ROI receiving field strengths over 2.25 V/cm and the percentage for which TER > 1 as primary outcome measures. Since we expect lower field strengths (above 1.10 V/cm) to also have an inhibitory effect on tumor growth, we additionally provide results for field strengths above 1.50 V/cm and TER > 0. Figure 1 shows optimization curves that display the minimum required current for various percentages of ROI volume with $\mathrm{TER}>0$ or $\mathrm{TER}>1$. Supplementary Figure S1 shows similar curves for field strength and Supplementary Table S1 presents statistics. Transcranial TTFs were the most effective (i.e., highest percentage reached) for the temporal and least effective for the brainstem tumor, while intracranial TTFs were the least effective for the thalamic tumor. Using 0.9 A current (the clinically employed value for conventional TTF), transcranial TTFs reached $\mathrm{TER}>1$ in 2% of the ROI volumes examined and $\mathrm{TER}>0$ in 8–53% across al ROIs while intracranial TTFs achieved $\mathrm{TER}>1$ in 98–100%, and $\mathrm{TER}>0$ in 100% of the ROIs for all three tumor cases. Interestingly, achieving $\mathrm{TER}>0$ in 50% of the ROIs examined required 7–15 times as much current with transcranial as compared to intracranial TTFs.

**Figure 2:**
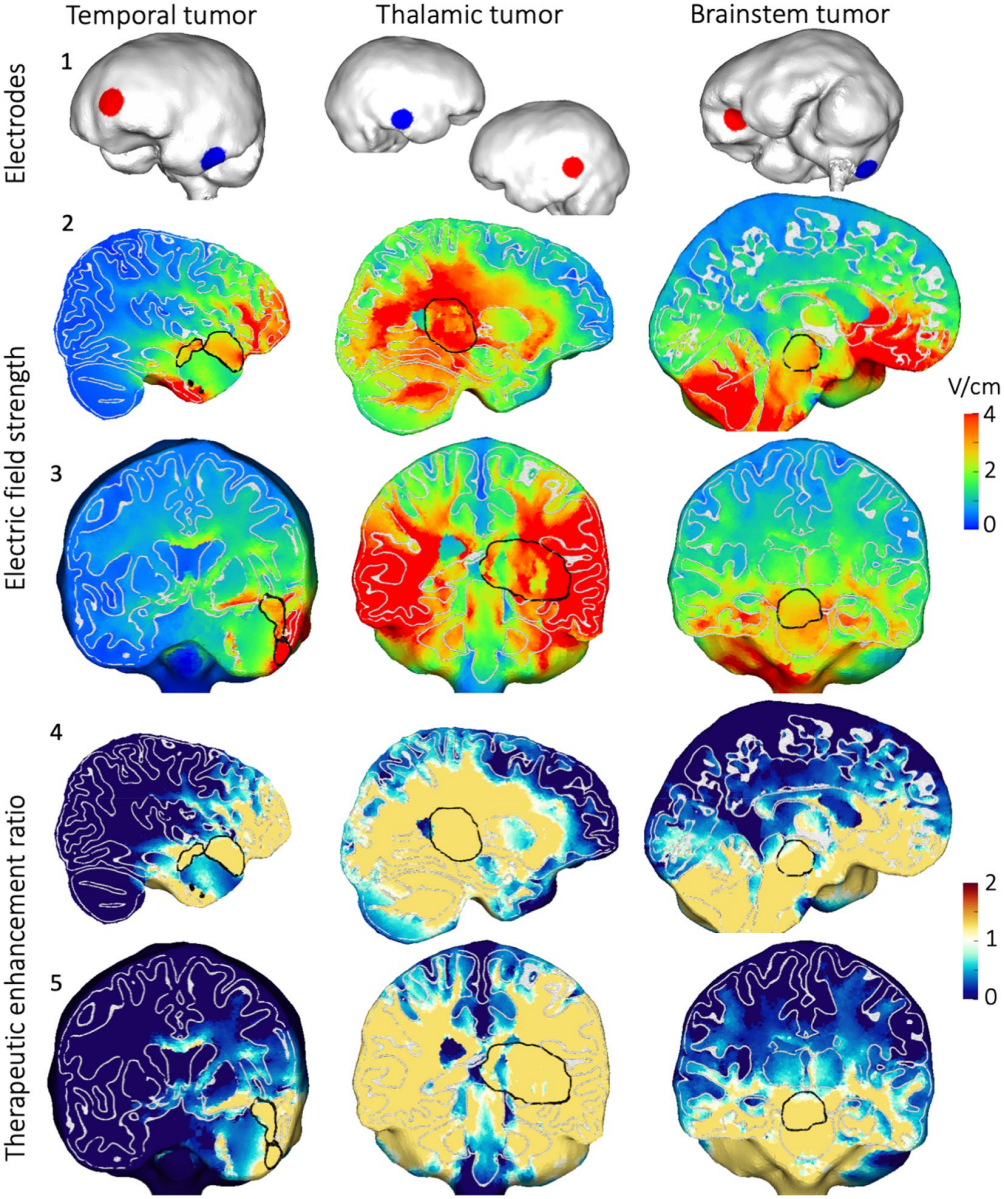
Optimal intracranial configurations and results. The optimal configurations (row 1) produced electric fields in the brain (rows 2–3: sagittal and coronal views) that achieved a TER in the tumor (rows 4–5: identical views). Results are shown on cuts through the center of the tumor, which is outlined in black. Gray and white matter boundaries are outlined in white. The selected configurations achieved $\mathrm{TER}>1$ in at least 90% of the ROI (marked in Fig. 1; details in Supplementary Table S2).

To examine the electric field and TER distributions throughout the brain, we selected one “optimal” configuration for each case; this was defined as the configuration that required the minimum current to achieve $\mathrm{TER}>1$ in at least 90% of the ROIs (marked with a cross in Fig. 1); such configurations are expected to have high clinical efficacy without needing to maximize the injected current. The optimal intracranial configuration for the left temporal tumor consisted of one electrode along the left anterior orbitofrontal cortex and one electrode on the posterior left temporal lobe (Fig. 2, top row). Of note, electrodes close to the ROI and on opposite sides appear to produce the largest electric fields in a superficial ROI. The optimal configuration for the thalamic tumor consisted of one electrode on the left temporal-parietal cortex and one on the right temporal cortex, resulting in current that flowed roughly through the center of the brain (Fig. 2, top row). These electrodes were farther away from the ROI than for the temporal tumor and were in both hemispheres, thereby directing the current to a central ROI. The optimal brainstem tumor consisted of one electrode on left orbitofrontal cortex and one on right posteroinferior cerebellum above the foramen magnum, resulting in an anteroposterior current flow on the inferior brain surface (Fig. 2, top row).

The electric field in the brain was much more focal for the temporal tumor than for the other two cases (Fig. 2, rows 2–3) due to the ROI being relatively superficial, resulting in electrodes that were in close proximity. Histograms of field strengths within the ROI are shown in Supplementary Fig. S2A. Critically, as compared to the optimal transcranial results (Supplementary Fig. S3), the intracranial models produced stronger and more focal fields.

Rows 4–5 of Fig. 2 show TER on the same cross planes: all yellow areas reached $\mathrm{TER}>1$ and as such would be expected to achieve proliferation arrest and/or tumor shrinkage in clinical practice. Importantly using our intracranial configurations this was achieved in majority of the tumor ROIs for each of the three cases examined. While the thalamic tumor core was projected to receive lower field strengths than the shell/surrounding tissue due in part to the core’s higher conductivity most of the core did reach clinically relevant $\mathrm{TER}>0$. Histograms of TER values within the ROIs for the intracranial configurations were similar with large peaks having been noted for $\mathrm{TER}=1$ (Supplementary Fig. S2B); as evidenced by the distributions in the temporal and thalamic and brainstem ROIs, transcranial stimulation was much less effective at achieving clinical relevant TERs (Supplementary Fig. S2B).

When providing a single “optimal” configuration, it is prudent to note that one of the electrode locations may in fact be difficult to reach surgically. The benefit of the optimization approach used here is that we can also provide multiple “near-optimal solutions” (Supplementary Table S2). The differences in current required between the first and fifth solutions are 0.9–3.8% across the three cases, demonstrating that multiple configurations can essentially achieve the same effects with similar amounts of current. For the temporal and thalamic cases, each of the top five configurations has one electrode in common, while for the brainstem case, there is more variety. As we think about advancing such an approach clinically, if for a specific patient, one of these optimal electrode locations could not be reached surgically, we could ultimately remove that electrode from the set and repeat the optimization.

As discussed above, electrodes were modeled as 10-mm disks based on the conventional Novocure TTF array. Intracranial cortical stimulation for other purposes is generally done using grids of small circular electrodes. Using an array of smaller electrodes will affect the resistance of the system and therefore can change the intensity of the fields. To investigate these effects, we replaced the optimal electrodes from Fig. 2 with 2 × 2 arrays of 3-mm radius electrodes (Supplementary Fig. S4) and simulated TTF with the same amount of current spread across the electrodes of each array. The resulting distributions of electric field strength and TER throughout the brain strongly resemble those for stimulation with single large electrodes (Supplementary Fig. S4 vs Fig. 2), however, field strengths are higher when using the 2 × 2 arrays as was the percentage of tumor volume receiving $\mathrm{TER}>1$.

## Discussion

Recent evidence has come to suggest that higher electric field strengths result in more effective inhibition of tumor growth with resultant improvements in overall survival in GBM^6–8,13^. While the dose response curves used to estimate rates of tumor inhibition rely on in vitro data, it is accepted that field intensity predicts the degree of tumor inhibition as a measure of treatment dose^13^. Notably, a recent phase I study of skull remodeling in recurrent GBM was the first to demonstrate prospectively that higher field strengths correspond with longer median survival^13^. A second important component of treatment dose is the amount of time that the therapy is delivered, and as such it is perhaps unsurprising that extending daily treatment duration contributes to improved survival^8,10^. Our results demonstrate the potential for substantial improvement in the TTF dose using an implanted stimulator by improving both strength and duration of TTF delivery.

Due to anatomic and biophysical constraints, transcranial stimulation with Novocure’s existing Optune^®^ device provides a variable degree of therapeutic field intensity to treatment area(s) within the brain but as evidenced above is generally unable to reach doses associated with complete tumor arrest in large portions of the tumor volume. It is also prudent to note that unlike radiation, which may affect the entire tumor volume, TTFs are likely to have highly heterogeneous field strengths within the tumor due to variations in conductivity, proximity to CSF spaces, electrode location and/or anatomic variations such as skull thickness and tumor depth^15–17^.

Tumor and treatment heterogeneity have substantial implications, and conceptually, the inability to reach therapeutic intensities within an entire tumor bed is in some ways functionally akin to a subtotal resection, with a larger untreated percentage of tumor conferring worse clinical outcomes^18,19^. Surgery remains a mainstay of GBM therapy, and gross total resection confers a survival advantage during initial surgery and at the time of recurrence^20–24^. A similar phenomenon might logically exist in TTF, such that treating nearly 100% of the tumor volume or peri-resection region with high intensity fields may confer a significant survival benefit. Accordingly, the authors posit that using intracranial electrodes may provide a modality to accomplish such a goal.

As surgery is already part of the standard of care for GBM, neurosurgeons are heavily involved in the care of such tumor patients. In addition to resective surgeries, several experimental immunotherapies rely on neurosurgical delivery of the therapeutic agent to the tumor bed^25–27^. The addition of another surgical modality into the therapeutic arsenal targeting GBM would therefore likely be widely accepted by practitioners in both neurosurgery and neuro-oncology.

Certain challenging cases, such as thalamic or brainstem GBMs, are often unresectable due to the involvement of eloquent and/or critical brain structures. In such cases, prognosis is even worse, with median survivals of only 12.2 months having been documented in patients with thalamic GBM^28^, 6 months for brainstem GBM^29^, and approximately 12 months for pediatric diffuse intrinsic pontine glioma (DIPG)^30^. As we have demonstrated despite attempts at optimization of electrode placement and skull reduction surgery, tumors located deep within the brain remain elusive targets for transcranial TTFs. Such cases are among the most compelling examples of where intracranial TTF might be of immediate clinical benefit. While no such device exists at this time, Novocure has recently recognized the potential benefits of an implanted TTF device^31^. Despite the significant potential of existing transcranial TTF therapy, adoption remains infrequent among patients with GBM and their treating physicians. In addition to the critiques over trial design, others have emphasized a “hassle” factor due to frequent head shaving, daily device placement, and the visibility of a device associated with brain cancer treatment, which may result in decreased quality of life. Concerns about patient convenience and compliance were considered substantial barriers to use in a recent survey of medical and neuro-oncologists^32^. When paired with what has been considered a relatively modest increase in overall survival, these obstacles have proved difficult to surmount^32,33^. An intracranial approach may overcome several of these barriers; with an implanted system, patients would no longer need to shave or apply large electrode pads to the scalp. In addition, a fully implanted system could function similarly to a conventional deep brain stimulation system and run up to 24 h per day, if desired, all of which should result in improved compliance and ultimately clinical outcomes. Such an approach may also serve an adjuvant treatment modality for to a litany of other emerging experimental therapies (e.g., immunotherapy)^34^. Lastly, an implanted array could potentially drive innovation in the growing field of cancer neuroscience, for example creating a platform that might enable physiologic recording within and/or adjacent to a tumor^35^.

Obstacles to implementation of such an approach are primarily related to the specifics of energy delivery. We have demonstrated the relationship between current delivered and the electric field strength in the targeted tumor. While higher currents will result in higher field strengths, cortical stimulation at 200 kHz has not been tested in vivo*,* and we therefore cannot know the safe upper limit for therapeutic stimulation of this type when applied to the dura or cortical surface. Traditional estimates of tissue damage thresholds during macro-electrode neurostimulation have compared stimulation intensity to damage thresholds from in vivo experiments, such as the Shannon equation^36^ A charge density limit was established for most intracranial electrodes at 30 μC/cm^2^, with the exception of the much higher charge densities allowed in extradural spinal cord stimulation, where high-intensity stimulation is mitigated by the dura and CSF. Given the limited parameters for pulse width, current and electrode area used in the Shannon equation, emerging forms of stimulation may not conform to these established thresholds^37–41^. Furthermore, all of these studies evaluated pulsed stimulation, which differs fundamentally from the higher-frequency alternating current delivered using TTFs.

The Optune device delivers current with an amplitude of up to 0.9 A but not all of it reaches the brain given diffusion into the scalp and the insulating effects of the skull. If we assume a 75% loss^42^, the current reaching the brain is 2.4–2.8 times lower than the 0.54–0.64 A needed for our intracranial models to achieve growth arrest in the complete tumor, or approximately 0.225 A. If we inject 0.225 A intracranially we can achieve growth arrest in 14–96%, and growth reduction in 90–100%, of the ROIs, as compared to 2% and 8–52% with 0.9 A transcranial TTF. It is prudent to note that this approximation is reductive, does not control for other important variables such as number of electrodes and size of electrodes in the array, and does not replace the necessity for in vivo tissue injury experiments. It does however offer initial evidence that the stimulation parameters we are proposing are likely to be safe and compare favorably with the stimulation intensity delivered via approved treatment methods.

The high energy requirement for TTF would clearly be reduced by using an intracranial electrode array, but our data suggest that some intracranial arrays may use more energy than can be provided by current commercially available implantable medical batteries. We hope that battery technology will advance to allow for safe, fully implantable batteries of this capacity, but viable alternatives allow for more immediate implementation. While not modeled in this work, electrodes placed within or immediately around a tumor volume might further reduce the energy requirements, facilitating an implantable system. In cases where life-saving devices require high energy requirements, precedent does exist for the use of an external power supply with an implanted device, with ventricular assist devices as a prime example^43^. Of course, this solution comes at the cost of higher infection rates^44^, and should be reserved for technologies with significant life-saving potential and no implantable alternative.

One additional consideration important for clinical implementation is the application of fields in multiple directions. Cells are more affected by TTF when mitotic spindles are directionally aligned with the electric fields, and the interleaved application of fields in multiple directions has been shown to increase the strength of tumor inhibition in both cell culture and animal models^6,7^. The Optune system does use two sets of electrodes to capitalize on these findings. In our model, we evaluated all possible configurations of intracranial electrode pairs and a comprehensive set of transcranial pairs. When designing a clinical array, a set of two roughly perpendicular pairs could be selected from all modeled pairs using the optimization methods described here.

## Conclusion(s)

The reconceptualization of TTF as a targeted, invasive therapy has the potential to provide a meaningful survival benefit to all patients with GBM and other brain tumors, even those with unresectable or surgically challenging tumors. The combination of in vitro dose response curves, clinical data corroborating the impact of higher TTF doses on improved survival, and the in silico evidence presented here should be sufficient to warrant the development and testing of an intracranial TTF stimulator. Research in TTF will benefit tremendously from an ongoing multi-disciplinary approach; neuro-oncologists and surgeons should work closely with engineers and computer scientists to optimize the therapy and hopefully substantially improve outcomes for patients with GBM.

## Materials and methods

Via the finite element modeling (FEM) of intracranial versus transcranial electrodes we investigated TTFs for three surgically complex GBM cases. Tumors were segmented from patient scans and built into a detailed base head model to allow for comparison between cases without the confounding effects of individual head geometry. Electrodes were built into the models’ skin (transcranial) or CSF (intracranial) surface and simulations were performed for a comprehensive set of electrode configurations and current amplitudes. Finally, optimal stimulation parameters were selected for transcranial and intracranial TTFs and in so doing the theoretical treatment outcomes were compared.

### Head models

**Figure 3:**
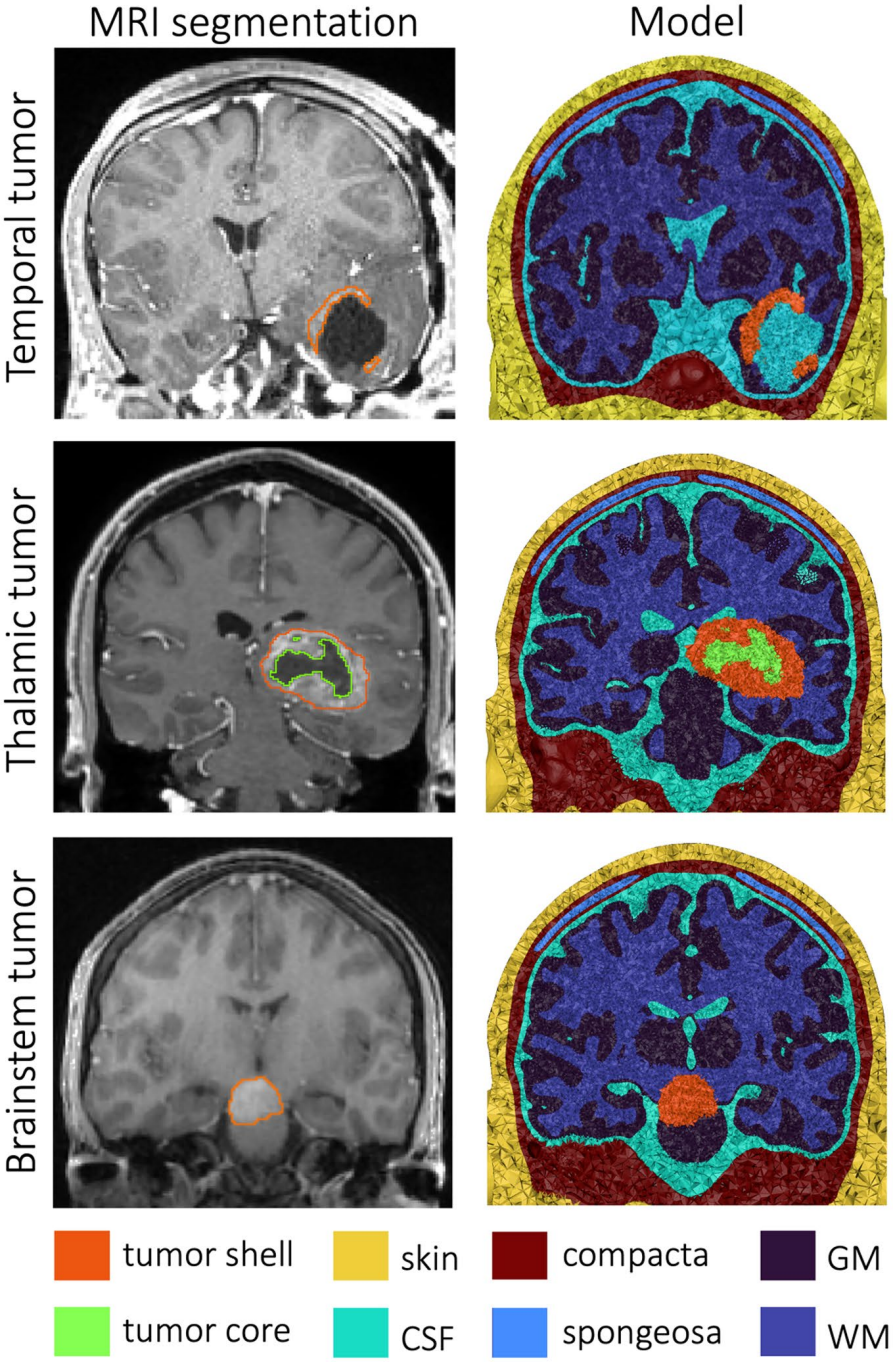
Tumor head models. For three tumor cases, tumors were segmented from patient MRIs (left) and the segmentation masks (outlines on the MRIs) were mapped onto the base model, resulting in three head models (right).

A finite element model was generated from T1-, T2- and diffusion-weighted MR images of a healthy 25-year-old man, as previously described^45^. Conductivity tensors were calculated using the volume-normalized approach^46^ and multiplied with the effective conductivity values for GM (0.276 S/m) and WM (0.126 S/m). All other compartments were assigned isotropic conductivities: skin (0.465 S/m), skull compact bone (0.007 S/m), skull spongy bone (0.025 S/m), CSF (1.65 S/m), eye (1.5 S/m), muscle (0.4 S/m)^45^. For simulations of intracranial TTFs, tissue compartments outside of the CSF were removed for ease of modeling, as has been done in electrocorticography simulations^47^. Removing these superficial tissue layers may result in a slight overestimation of the intracranial field strengths; however, in practice, we expect these differences to be minimal, given the presence of a silicone insulating layer on most intracranial electrodes, and the substantially lower conductivity of skull when compared to CSF and brain tissues. We selected three representative HGG cases treated at Brigham and Women’s Hospital between 2018 and 2020; informed consented was acquired. Evaluation of patient data was approved by the Mass General Brigham Institutional Review Board (IRB); data/experiments were collected/conducted in accordance with pertinent National Institutes of Health (NIH) guidelines. The three representative tumors selected were as follows: a left temporal GBM status post subtotal resection, a thalamic GBM, and an isolated HGG within the brainstem. T1 images of each patient and the base head model were skull stripped using the FSL bet program^48,49^, followed by registration of patient scans to the model scan using a linear transform in 3DSlicer^50^. The tumor shell (all cases), core (thalamic tumor) and resection area (temporal tumor) were segmented from the registered scans in Seg3D^51^ (Fig. 3, column 1). The segmentation masks were then mapped onto the base model using SCIRun^52^ (Fig. 3, column 2). The tumor core (1.0 S/m) and shell (0.24 S/m) were given different conductivities and the resected area was assigned the conductivity of CSF^53^.

### Electrode configurations

**Figure 4:**
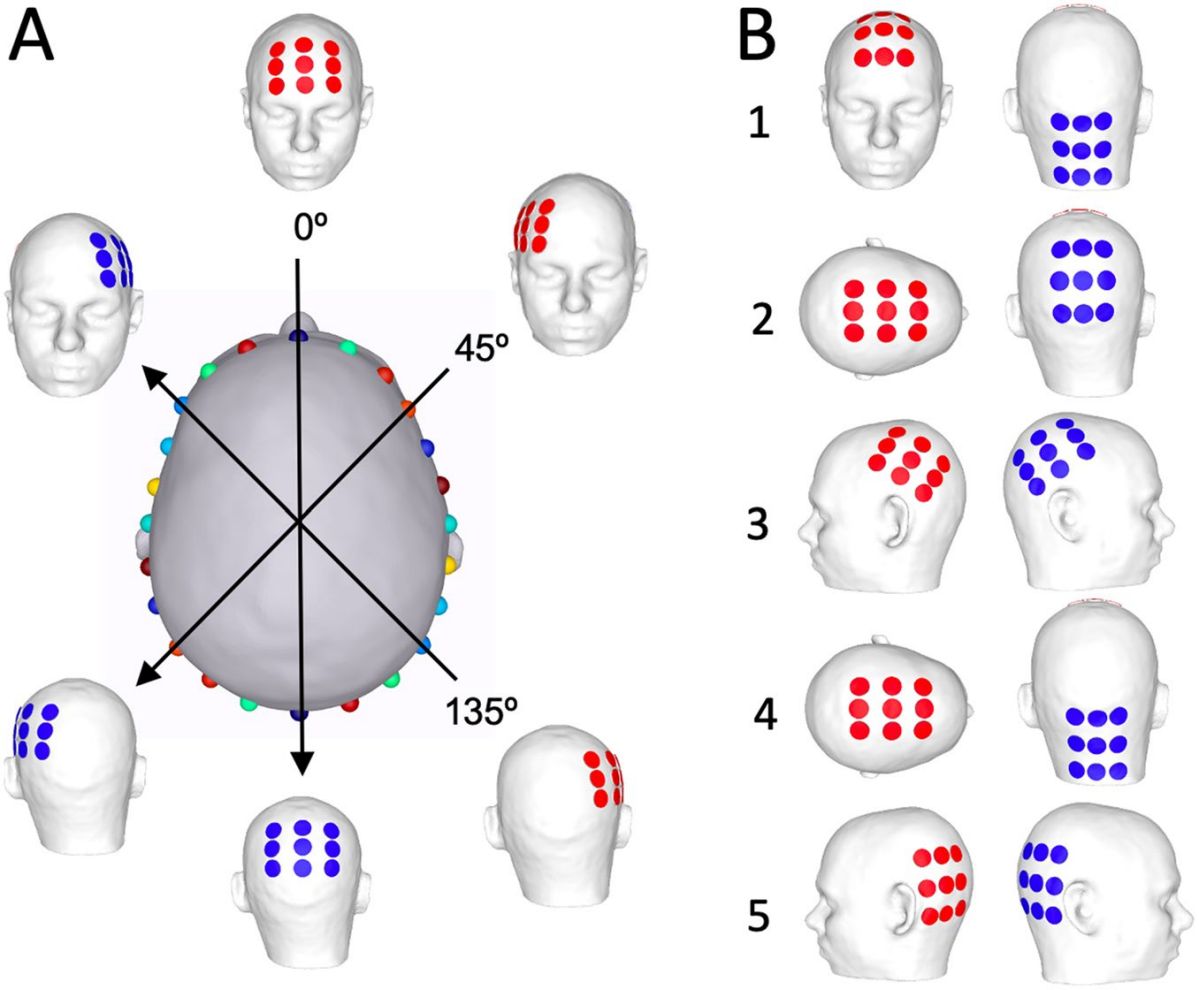
Transcranial electrode design. (**A**) The center image shows the transcranial model from above. We started on the line marked 0°; two patches were centered on the locations indicated by the blue spheres, resulting in the model shown at the top (anterior view) and bottom (posterior view) of the line. This pair of patches was then rotated around the vertical axis; spheres on the center image indicate the patch centers. Examples are shown at 0°, 45° and 135°. (**B**) Five transcranial configurations were based on prior studies.

Transcranial: We used a Novocure 3 × 3 electrode patch with 22 (vertical) and 33 (horizontal) mm respectively between electrode centers, an electrode radius of 10 mm and height of 2.5 mm. Electrodes were placed on the skin surface in an anteroposterior configuration similar to Novocure’s proscribed recommendations^54^. The pair of patches was rotated around the vertical axis of the head in 15-degree steps, ultimately producing 12 configurations (Fig. 4A), as was similar to a previous optimization study^15^. We also recreated all configurations from prior modeling studies (Fig. 4B): (1) anteroposterior with lower posterior electrode^54^, (2) patches on the top and back of the head^55^, (3) patches above the ears^55^; and two configurations proposed for infratentorial stimulation by Novocure^56^: (4) patches on the top of the head and the neck, (5) patches behind the ears. The complete set comprised 17 models in total.

**Figure 5:**
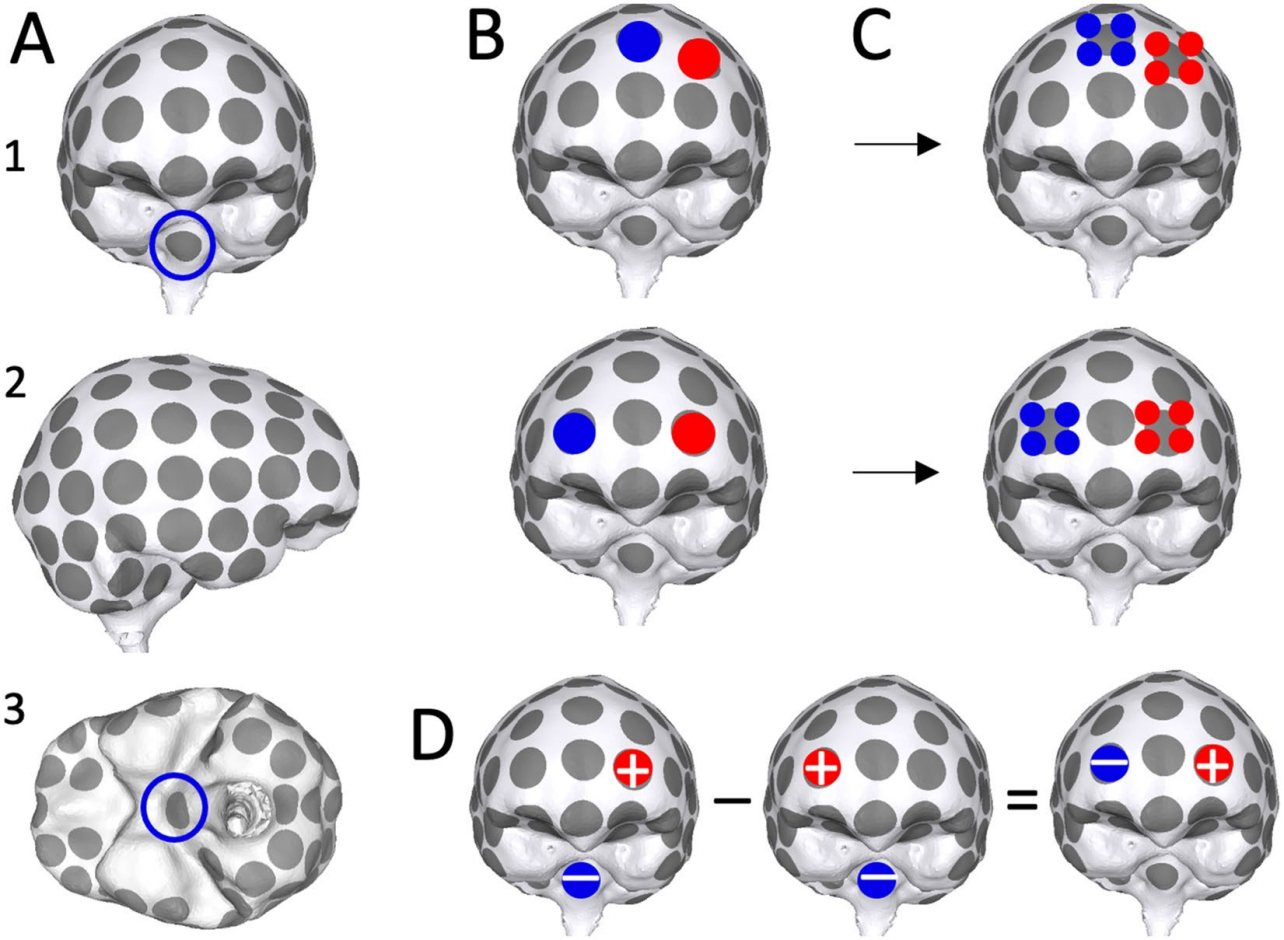
Intracranial electrode design. (**A**) Anterior (1), right (2) and inferior (3) views of the intracranial model; 101 electrodes were spread across the CSF surface and a reference (necessary for simulations, but not clinical practice) was placed on the brainstem (marked with blue circles). Supplementary Fig. S1 presents additional views. (**B**) Two examples of 2-electrode configurations. (**C**) Each electrode in the simulated two-electrode configurations can be replaced with small-electrode arrays. (**D**) Electric fields for each electrode pair were calculated by simulating each electrode with the reference (first two images), and then taking the difference of the two (result on the right).

Intracranial: To our knowledge there is no precedent for intracranial TTF, so the ideal size, number and placement of electrodes within such an array remains unknown. We therefore used pairs of 10-mm electrodes as placeholders allowing for optimization of locations without assumptions with regard to size and/or numbers. We placed 61 electrodes on the model’s CSF surface based on the 10–10 electrode system and added 40 electrodes below (Fig. 5A). Electrodes were not placed along the interhemispheric space or abutting the tentorium given that these locations are difficult to reach surgically. One reference electrode was placed in an area not intended for stimulation. There are $\frac{101!}{2! (101-2)!}=5050$ ways to take 2 out of 101 electrodes, each of which was simulated (two examples in Fig. 5B). Of note, once the optimal locations are identified, smaller electrodes can replace larger ones if so desired (two examples in Fig. 5C).

### Simulations

For each transcranial configuration, the quasistatic approximation to Maxwell’s equations was solved using SCIRun 4.7^45,52^. Intracranial configurations were simulated by first solving for each of 101 electrodes paired with the reference, and then taking linear combinations of the resulting electric fields (example in Fig. 5D).

### Predicted clinical efficacy

**Figure 6:**
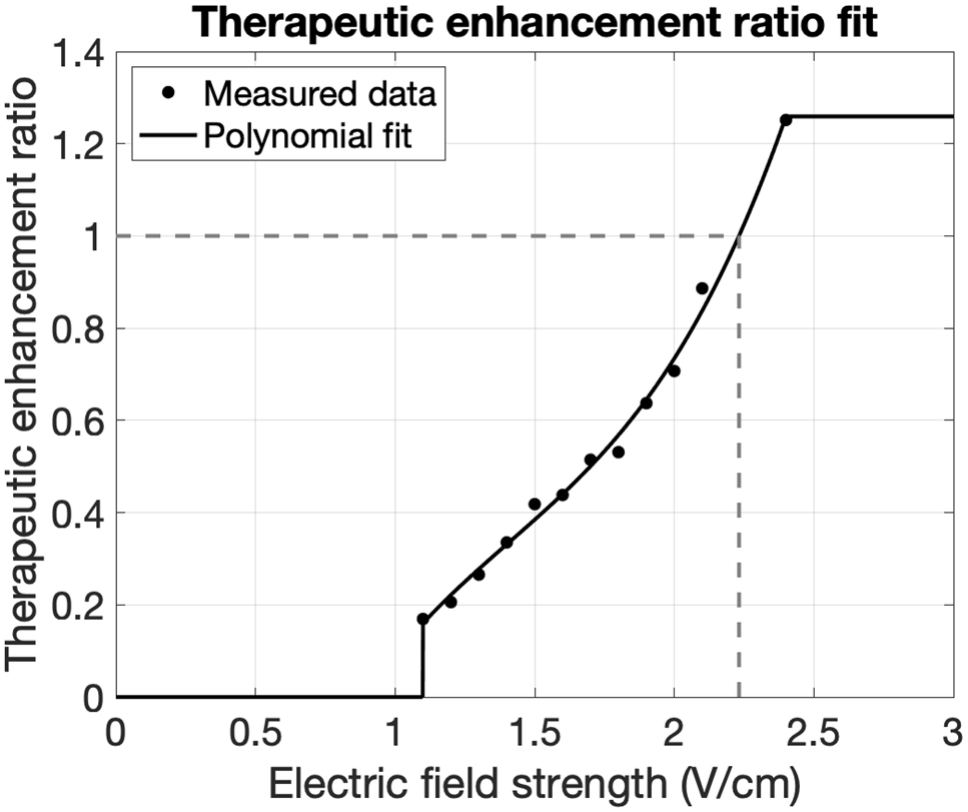
Fit of in vitro TER data. Black dots are a reproduction of data from Kirson et al.^6^ who applied TTF to malignant glioma cell cultures and measured TER values. We fit a polynomial (black line) to the experimental data and capped values outside the reported range. $\mathrm{TER}=1$ indicates complete growth arrest, which happens at *E* = 2.23 V/cm (dashed line). This differs slightly from prior work (2.25 V/cm^14^) due to a small difference in the fit.

As discussed above, the therapeutic enhancement ratio (TER) quantifies how electric field strength (*E*) affects tumor growth with Kirson et al. having reported TER values for tumor cell cultures resulting from the application of electric fields of various strengths^6^. Similar to the procedure described by Korshoej et al.^14^ we fitted a third-degree polynomial to the in vitro data: $\mathrm{TER}(E) =0.4057\times {E}^{3}-1.713\times {E}^{2}+2.941\times E-1.542$ (Fig. 6). Data was available for *E* values from 1.10 to 2.40 V/cm, so we set TER for *E* lower than 1.10 V/cm to 0, and for *E* higher than 2.40 V/cm to the value at *E* = 2.40 V/cm: $\mathrm{TER}(2.40) = 1.26$ (Fig. 6). For each simulated electric field, we used this function to calculate TER in each element of the model. TER values in the tumor were then used to find electrode configurations that maximized the tumor volume for which $\mathrm{TER}>0$, which corresponds to reduced tumor growth, or $\mathrm{TER}>1,$ which corresponds to growth arrest.

### Optimization

We performed a Pareto optimization that balanced the injected current and TER achieved in the regions of interest (ROI) within our models. The ROI was defined as all tumor tissue, which for the thalamic tumor included the core and shell. Negative effects from stimulating areas outside of the ROI have not been reported in clinical studies, so we focused on maximizing effects in the ROI and did not consider focality. For each configuration, electric fields were calculated for 2000 values of injected current linearly distributed between 0 and 2 A. For each solution, the percentage of ROI volume for which the $\mathrm{TER}>0$, or $\mathrm{TER}>1$, was calculated. The solutions were then grouped based on their percentage into bins between 0 and 100%; in each bin, we selected the configuration that required the least amount of current to achieve that percentage. Because lower field strengths may still have some therapeutic relevant effects, and higher field strengths are expected to have a greater effect, we repeated this analysis for field strengths between 1.0 and 3.0 V/cm.

## Supplementary Information

Supplementary Information.

## Data Availability

All data is available on request from the corresponding authors (DJS, JDB).
