# Author Correction: Modeling of intracranial tumor treating fields for the treatment of complex high-grade gliomas

**DOI:** 10.1038/s41598-023-30970-9

**Published:** 2023-03-09

**Authors:** David J. Segar, Joshua D. Bernstock, Omar Arnaout, Wenya Linda Bi, Gregory K. Friedman, Robert Langer, Giovanni Traverso, Sumientra M. Rampersad

**Affiliations:** 1 Department of Neurosurgery, Brigham and Women’s Hospital, Harvard Medical School, Boston, MA USA; 2 David H. Koch Institute for Integrative Cancer Research, Massachusetts Institute of Technology, Cambridge, MA USA; 3 Department of Pediatrics, University of Alabama at Birmingham, Birmingham, USA; 4 Department of Mechanical Engineering, Massachusetts Institute of Technology, Cambridge, MA USA; 5 Division of Gastroenterology, Brigham and Women’s Hospital, Harvard Medical School, Boston, MA USA; 6 Department of Physics, University of Massachusetts, Boston, MA USA; 7 Department of Electrical and Computer Engineering, Northeastern University, Boston, MA USA

Correction to: *Scientific Reports* https://doi.org/10.1038/s41598-023-28769-9, published online 30 January 2023

The original version of this Article omitted affiliations for Giovanni Traverso. The correct affiliations are listed below.

David H. Koch Institute for Integrative Cancer Research, Massachusetts Institute of Technology, Cambridge, MA, USA.

Department of Mechanical Engineering, Massachusetts Institute of Technology, Cambridge, MA, USA.

Division of Gastroenterology, Brigham and Women’s Hospital, Harvard Medical School, Boston, MA, USA.

In addition, the Author Contributions section contained an error.

“Conception and design: D.S., J.B., S.R. Data acquisition: D.S., J.B. Analysis and interpretation of data: D.S., J.D.B., S.R. Study supervision: O.A.R., W.L.B., G.F.K., R.S.L., G.T. Critical drafting/revision of manuscript: all authors.”

now reads:

“Conception and design: D.S., J.D.B., S.R. Data acquisition: D.S., J.D.B. Analysis and interpretation of data: D.S., J.D.B., S.R. Study supervision: O.A.R., W.L.B., G.F.K., R.S.L., G.T. Critical drafting/revision of manuscript: all authors.”

The original Article has been corrected.

