# Supplementary material for: Modeling of intracranial tumor treating fields for the treatment of complex high-grade gliomas
Source: Sci Rep. 2023 Jan 30;13:1636. doi: 10.1038/s41598-023-28769-9 (PMC9886948; doi:10.1038/s41598-023-28769-9)
Supplement: Supplementary file 1 — Supplementary Information. [file 41598_2023_28769_MOESM1_ESM.pdf]

SUPPLEMENTAL FIGURES

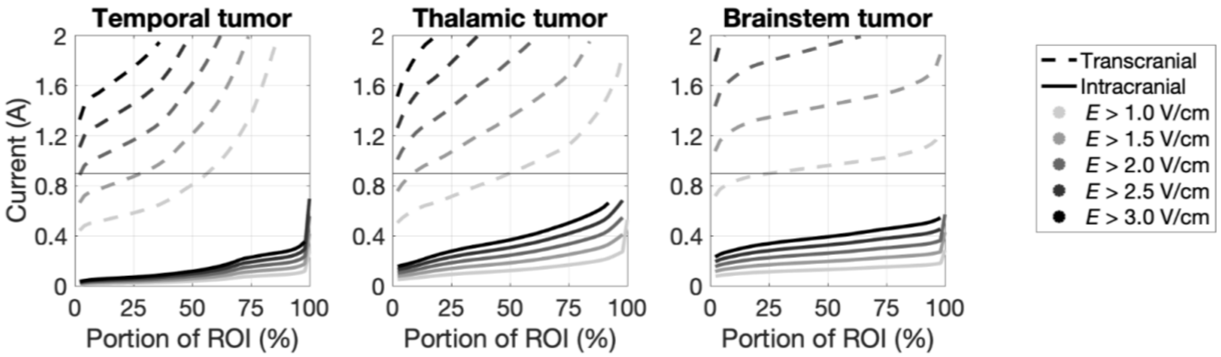

Figure S1. Electric field strength optimization curves for transcranial and intracranial TTF. Each curve indicates the amount of current needed to achieve a certain electric field strength (E) in a given percentage of the ROI volume. For example, the first panel presents the results for the temporal tumor; the dashed black line (top most line) shows that with an optimized transcranial configuration, one can reach a field strength of at least 3.0 V/cm in 25% of the ROI volume using 1.8 A of current. Horizontal lines indicate the current level conventionally used for transcranial TTF (0.9 A). The amount of current needed to achieve a certain field strength in a given percentage of the ROI increases linearly with the desired field strength. For the brainstem tumor, 3.0 V/cm is not reached in any part of the ROI, hence there is no corresponding curve.

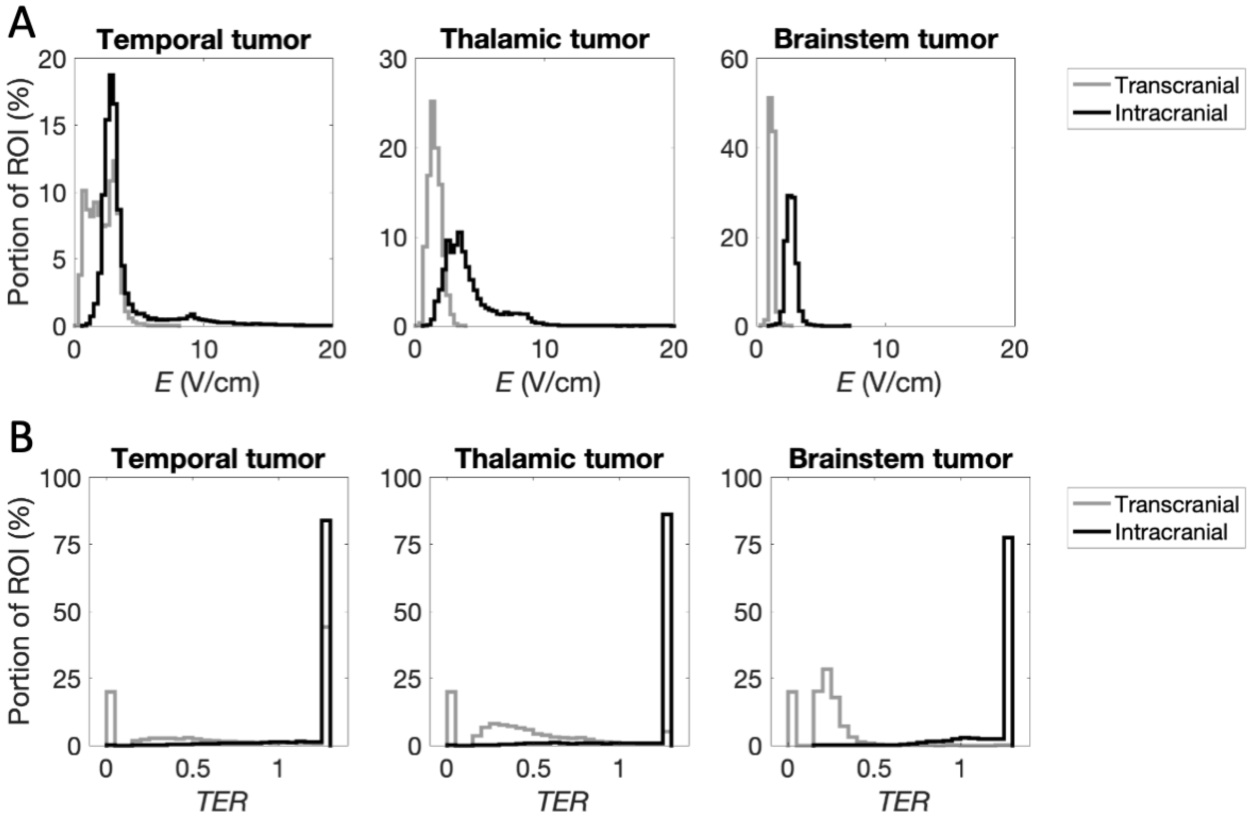

Figure S2. Results in the ROI for the optimal configurations for transcranial and intracranial TTF. The optimal configurations were defined as those that achieve TER > 0 for transcranial TTF, or TER > 1 for intracranial TTF, in at least 90% of the tumor volume with the minimum amount of current needed to achieve this. These configurations are marked with a cross in Fig. 5. (A) Histograms of electric field strength in the ROI. For intracranial stimulation, histograms peak around 3.0 V/cm for all cases, but the distributions in the temporal and brainstem ROIs are fairly narrow, while the thalamic ROI receives higher field strengths in a large portion of the ROI. With transcranial stimulation, peaks occur at lower field strengths. There is more overlap with the intracranial distribution for the temporal ROI, because it is more superficial and easier to reach transcranially compared to the other two. (B) Histograms of therapeutic enhancement ratio (TER) in the ROI. For intracranial stimulation, all distributions have a large peak at $TER = 1$, with a bump at slightly lower TER for the brainstem ROI. For transcranial stimulation, there is a much smaller peak at TER $= 1$ for the temporal and thalamic ROIs, while the brainstem ROI does not achieve TER $= 1$ anywhere. Each case also has a peak at TER $= 0$ and a bump at slightly higher values.

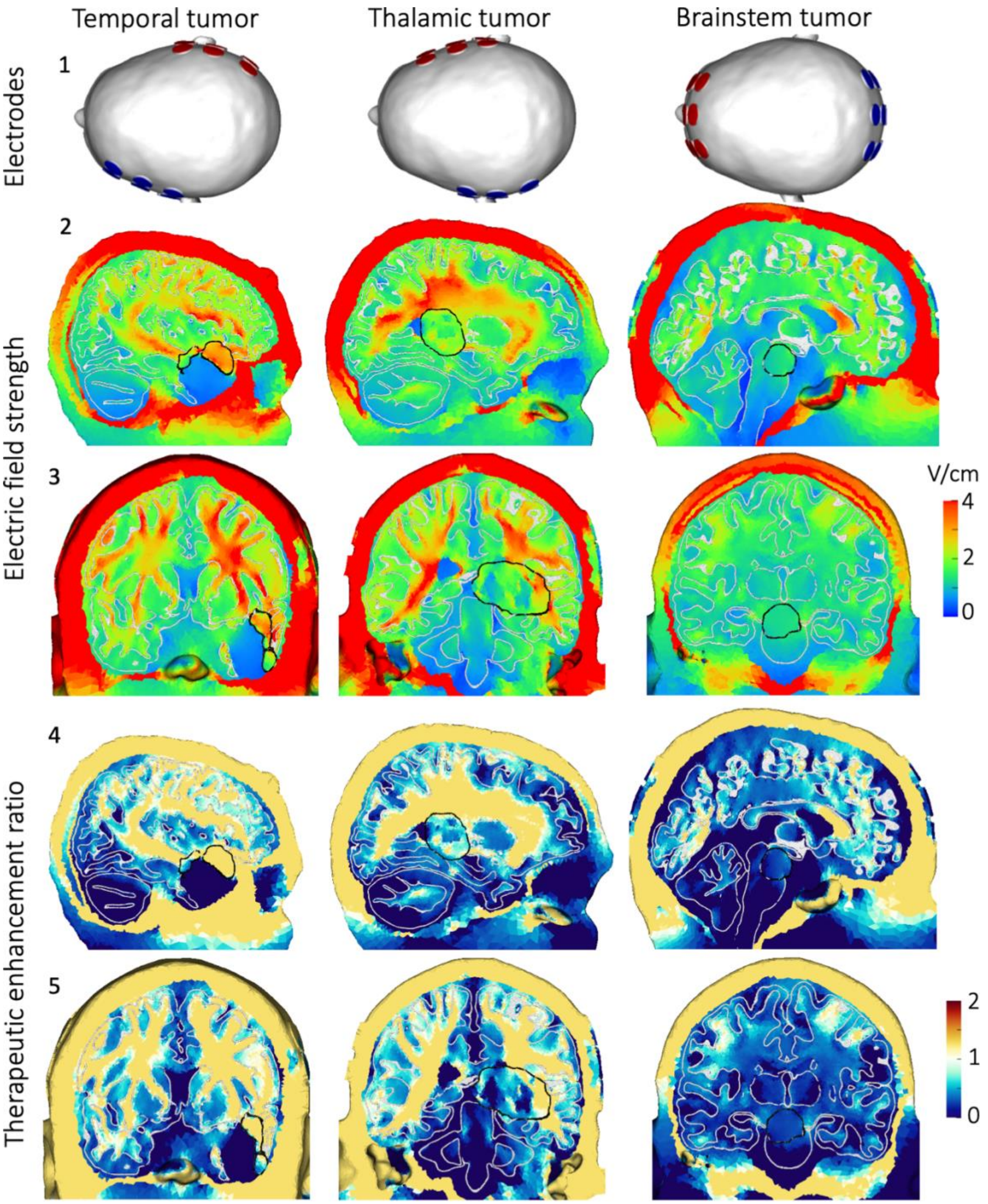

Figure S3. Optimal transcranial configurations and results. The optimal configurations (row 1) produced electric fields in the brain (rows 2–3: sagittal and coronal views) that achieved a therapeutic enhancement ratio (*TER*) in the tumor (rows 4–5: identical views). Results are shown on cuts through the center of the tumor, which is outlined in black. The cuts are identical to Fig. 2. Gray and white matter boundaries are shown in white. The selected configurations achieved $TER > 0$ in at least 90% of the ROI (modeled tumor) with the minimum amount of current needed to achieve this. These configurations are marked with a gray cross in Fig. 1.

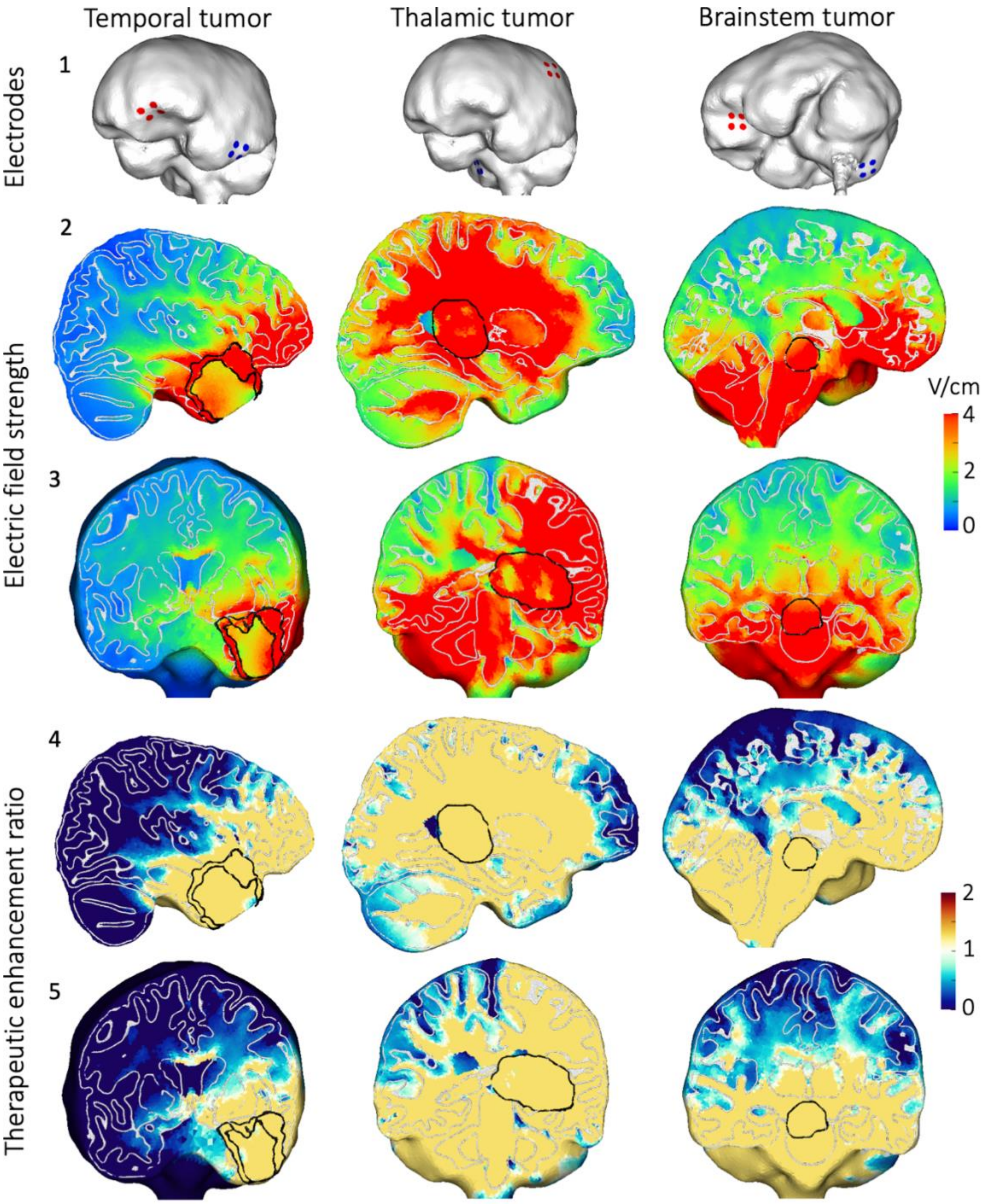

Figure S4. Optimized intracranial TTF using small electrodes. The optimal electrode configurations as shown in Fig. 2 were replaced with 2x2 arrays of small electrodes (row 1) and simulated with the same amounts of current (Table S2). This produced electric fields in the brain (rows 2–3: sagittal and coronal views) that achieved a therapeutic enhancement ratio in the tumor (rows 4–5: identical views). Results are shown on cuts through the center of the tumor, which is outlined in black. The cuts are identical to Fig. 2. Gray and white matter boundaries are shown in white.

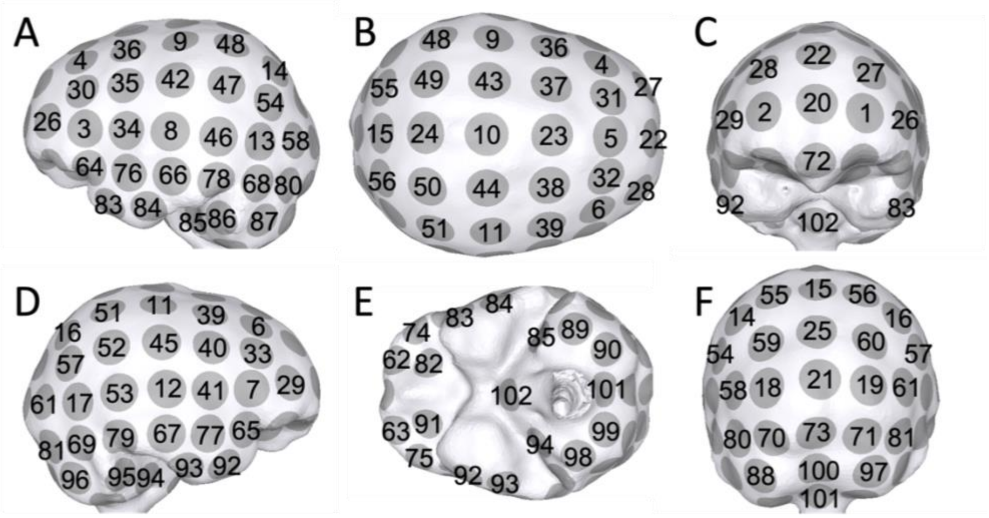

Figure S5. Electrode numbering for the intracranial model. Left (A), top (B), anterior (C), right (D), bottom (E) and posterior (F) views of the model's outer surface (i.e., the CSF surface) with electrodes embedded. These numbers correspond to the optimal configurations listed in **Supplementary Table S2.**

SUPPLEMENTAL TABLES

Table S1. Summary of results for optimization of TER and electric field strength for transcranial and intracranial TTF. The top half of the table presents, for each of three tumor cases, the percentage of ROI volume in which a certain TER value can be achieved given a desired amount of injected current, using either optimal transcranial or intracranial configurations. The bottom half of the table presents the minimum amount of current needed to achieve a certain TER or field strength in a desired percentage of the ROI volume.

Result	Method	Temporal	Thalamic	Brainstem
Portion of ROI reaching TER > 0 with 0.9 A injected current (conventional amount for transcranial TTF)	Transcranial	52%	40%	8%
	Intracranial	100%	100%	100%
Portion of ROI reaching TER > 1 with 0.9 A injected current	Transcranial	2%	2%	2%
	Intracranial	100%	98%	100%
	Transcranial	82%	96%	98%

Portion of ROI reaching TER > 0 with 2 A injected current (maximum amount simulated)	Intracranial	100%	100%	100%
Portion of ROI reaching TER > 1 with 2 A injected current	Transcranial	56%	48%	24%
	Intracranial	100%	100%	100%
Current needed to achieve TER > 0 in 50% of the ROI	Transcranial	0.89 A	0.99 A	1.06 A
	Intracranial	0.04 A	0.14 A	0.14 A
Current needed to achieve TER > 0 in 100% of the ROI	Transcranial	0.31 A	0.58 A	0.32 A
Current needed to achieve TER > 1 in 100% of the ROI	Intracranial	0.62 A	0.54 A<br>(98%)	0.64 A
Current needed to achieve E > [1.0, 1.5, 2.0, 2.5, 3.0] V/m in 50% of the ROI	Transcranial	0.81 A<br>1.21 A<br>1.62 A	0.9 A<br>1.35 A<br>1.8 A	0.96 A<br>1.44 A<br>1.92 A
	Intracranial	0.04 A<br>0.06 A<br>0.08 A<br>0.1 A<br>0.12 A	0.12 A<br>0.18 A<br>0.25 A<br>0.31 A<br>0.37 A	0.13 A<br>0.2 A<br>0.26 A<br>0.33 A<br>0.39 A

Table S2. Five best configurations for intracranial TTF for three tumor cases. For each case, the table presents the top five configurations that require the least amount of current to reach a therapeutic enhancement ratio (TER) greater than 1 in at least 90% of the ROI (the modeled tumor). For each configuration (each row), the table presents the required current amplitude, the percentage of the ROI that reaches TER > 1, and the numbers of the electrodes that make up that configuration. Electrode numbers are visualized on the model in **Supplementary Fig. S5**.

	**Temporal tumor**			**Thalamic tumor**			**Brainstem tumor**		
	**I (A)**	**TER>1 (%)**	**elec-trodes**	**I (A)**	**TER>1 (%)**	**elec-trodes**	**I (A)**	**TER>1 (%)**	**elec-trodes**
1	0.206	90.3	1, 84	0.469	90.0	46, 67	0.369	90.3	82, 99

2	0.206	90.2	27, 84	0.472	90.1	46, 79	0.378	90.2	82, 96
3	0.209	90.3	4, 84	0.472	90.0	46, 93	0.380	90.4	90, 91
4	0.213	90.3	62, 84	0.472	90.0	46, 94	0.382	90.1	91, 99
5	0.217	90.0	31, 84	0.473	90.1	12, 46	0.383	90.2	73, 82
